# A preliminary evaluation of antihyperglycemic and analgesic activity of *Alternanthera sessilis* aerial parts

**DOI:** 10.1186/1472-6882-14-169

**Published:** 2014-05-24

**Authors:** Ahamed Ismail Hossain, Mohammad Faisal, Shahnaz Rahman, Rownak Jahan, Mohammed Rahmatullah

**Affiliations:** 1 Department of Biotechnology and Genetic Engineering, University of Development Alternative, Dhanmondi, Dhaka 1209, Bangladesh; 2 University of Development Alternative, House No. 78, Road No. 11A (new),Dhanmondi, Dhaka 1209, Bangladesh

**Keywords:** Antihyperglycemic, *Alternanthera sessilis*, Glucose tolerance, Non-narcotic analgesic, Amaranthaceae

## Abstract

**Background:**

*Alternanthera sessilis* is used by folk medicinal practitioners of Bangladesh for alleviation of severe pain. The objective of this study was to scientifically analyze the analgesic (non-narcotic) property of aerial parts of the plant along with antihyperglycemic activity.

**Methods:**

Antihyperglycemic activity was measured by oral glucose tolerance tests. Analgesic (non-narcotic) activity was determined by observed decreases in abdominal writhings in intraperitoneally administered acetic acid-induced pain model in mice.

**Results:**

Administration of methanol extract of aerial parts led to dose-dependent and significant reductions in blood glucose levels in glucose-loaded mice. At doses of 50, 100, 200 and 400 mg per kg body weight, the extract reduced blood sugar levels by 22.9, 30.7, 45.4 and 46.1%, respectively compared to control animals. By comparison, a standard antihyperglycemic drug, glibenclamide, when administered at a dose of 10 mg per kg body weight, reduced blood glucose level by 48.9%. In analgesic activity tests, the extract at the above four doses reduced the number of abdominal writhings by 27.6, 37.9, 41.4, and 44.8%, respectively. A standard analgesic drug, aspirin, reduced the number of writhings by 31.0 and 51.7%, respectively, when administered at doses of 200 and 400 mg per kg body weight.

**Conclusion:**

The results validate the folk medicinal use of the plant to alleviate pain. At the same time, the antihyperglycemic activity result suggests that the plant may be a potential source for blood sugar lowering drug(s).

## Background

*Alternanthera sessislis* (L.) R. Br. (Amaranthaceae) is known in English as sessile joyweed or dwarf copperleaf and in Bangladesh as Chanchi shak. It is an aquatic plant and can be commonly observed in marshy areas and wetlands of Bangladesh. Folk medicinal practitioners of Bangladesh consider the plant to possess medicinal properties. In Noakhali district of Bangladesh, the plant is used to treat gonorrhea, low sperm count, and leucorrhea [1]. In several areas of Faridpur and Rajbari districts of Bangladesh, the plant is used by folk medicinal practitioners for treatment of severe pain [2]. The tribals of Bargarh district, India use the plant to treat blood dysentery [3]. Different communities of Uttara Kannada district of Karnataka, India use the plant for treatment of ulcers and cuts and wounds [4]. The plant is used by local tribals (Santals, Gonds, Kolha, Bathudi) and inhabitants of Kaptipada Forest Range in Orissa, India for treatment of fevers, ophthalmia, gonorrhea, and pruritis [5]. The local people of Amarkantak region, Madhya Pradesh India have multiple uses for the plant including treatment of burning sensations, diarrhea, skin diseases, dyspepsia, hemorrhoids, liver and spleen diseases, and fever [6]. The Irula tribals of Kalavai, Vellore district, Tamil Nadu, India, treat headache, hepatitis, and asthma with the plant [7].

Anti-bacterial activity and possible cytotoxicity as demonstrated by brine shrimp lethality assay has been reported for *A. sessilis*[8]. Ethyl acetate fraction of *A. sessilis* Red has been reported to reduce fasting blood glucose level, triglyceride level, and free fatty acid level when administered to obese type 2 diabetic rats induced by high fat diet and streptozotocin [9]. Anti-allergic effect of ethanolic extract of the plant has also been described [10].

Diabetes is a disease characterized by high blood glucose levels and is rapidly spreading throughout the world, possibly because of changes in lifestyle and dietary habits of people. The International Diabetes Federation (IDF) South East Asia (SEA) Region, which contains the countries of India, Sri Lanka, Bangladesh, Bhutan, Mauritius, and Maldives have a high prevalence of diabetes. It has been reported that there were more than 72 million adults with diabetes in 2013, a number which is expected to exceed 123 million by 2035. Nearly 95% people of the total diabetic patients have type 2 diabetes (T2DM). Moreover, another 24.3 million people have impaired glucose tolerance (IGT). Both rural and urban population has seen increases in the number of diabetic people [11]. In a cross-sectional survey of 402 people among the urban middle class in Bangladesh, it has been seen that 35% had T2DM and 45% had metabolic syndrome [12]. Diabetes can lead to other disorders like cardiovascular, kidney and eye disorders. In fact, a high prevalence of chronic kidney disease has been reported following a community survey of urban people of Bangladesh, which has been correlated with insulin resistance [13].

Pain is a feeling triggered in the nervous system and is a problem faced by people throughout the world on a daily basis. Pain can be acute (as caused through injury) or can be chronic (i.e. lasting for months or years, as is the case with patients suffering from rheumatoid arthritis, gout, or some forms of cancer). Over the counter (OTC) drugs like aspirin or paracetamol can cause gastric ulceration or hepatic damage from prolonged use or over-dosage.

Rural people of Bangladesh suffer from lack of access to modern doctors and health-care facilities. Moreover, they cannot afford the price of allopathic medicines, whether be it anti-diabetic or analgesic. As such, scientific validation of traditional remedies or scientific validation of a previously not known plant for any given pharmacological effect offers the rural people (with ready accessibility to the plant) to get a desired medicinal effect at a much cheaper cost. Since diabetes and pain are common illnesses in Bangladesh, we had been screening various plants of Bangladesh for possible antihyperglycemic and analgesic effects [14-16]. *A. sessilis* is a common aquatic plant of Bangladesh and is readily available and frequently consumed by rural people as a vegetable. The objective of the present study was to evaluate the antihyperglycemic and analgesic potential of methanolic extract of aerial parts of *A. sessilis* through oral glucose tolerance tests and acetic acid-induced gastric pain model, respectively in mice.

## Methods

### Plant material collection

Aerial parts (leaves and stems) of *A.sessilis* were collected during March 2013 from Mirpur in Dhaka district, Bangladesh and taxonomically identified at the Bangladesh National Herbarium (Accession Number 38,593).

### Preparation of methanolic extract of aerial parts

Aerial parts were cut into small pieces, air-dried in the shade, and 100 g of dried and powdered leaves and stems was extracted with methanol (w:v ratio of 1:6, final weight of the extract 8.03 g).

### Chemicals and drugs

Glibenclamide, aspirin, and glucose were obtained from Square Pharmaceuticals Ltd., Bangladesh. All other chemicals were of analytical grade.

### Animals

Swiss albino mice (male), which weighed between 15–19 g were used in the present study. The animals were obtained from International Centre for Diarrhoeal Disease Research, Bangladesh (ICDDR,B). The animals were acclimatized for three days prior to actual experiments. The study was conducted following approval by the Institutional Animal Ethical Committee of University of Development Alternative, Dhaka, Bangladesh.

### Oral glucose tolerance tests for evaluation of antihyperglycemic activity

Oral glucose tolerance tests were carried out as per the procedure previously described by Joy and Kuttan (1999) [17] with minor modifications. Briefly, fasted mice were grouped into six groups of five mice each. The various groups received different treatments like Group 1 received vehicle (1% Tween 80 in water, 10 ml/kg body weight) and served as control, Group 2 received standard drug (glibenclamide, 10 mg/kg body weight). Groups 3–6 received extract (MEAAS) at doses of 50, 100, 200 and 400 mg per kg body weight. All substances were orally administered. Following a period of one hour, all mice were orally administered 2 g glucose/kg of body weight. Blood samples were collected 120 minutes after the glucose administration through puncturing heart. Blood glucose levels were measured by glucose oxidase method [18]. The percent lowering of blood glucose levels were calculated according to the formula described below.

$$\mathrm{Percent}\;\mathrm{lowering}\;\mathrm{of}\;\mathrm{blood}\;\mathrm{glucose}\;\mathrm{level}=\left(1–W_{e}/W_{c}\right)\;\times \;100,$$

where W_e_ and W_c_ represents the blood glucose concentration in glibenclamide or MEAAS administered mice (Groups 2–6), and control mice (Group 1), respectively.

### Analgesic activity evaluation through abdominal writhing test

Analgesic activity of MEAAS was examined as previously described [19]. Briefly, mice were divided into seven groups of five mice each. Group 1 served as control and was administered vehicle only. Groups 2 and 3 were orally administered the standard non-narcotic analgesic drug aspirin at doses of 200 and 400 mg per kg body weight, respectively. Groups 4–7 were administered MEAAS at doses of 50, 100, 200 and 400 mg per kg body weight, respectively. Following a period of 60 minutes after oral administration of standard drug or MEAAS, all mice were intraperitoneally injected with 1% acetic acid at a dose of 10 ml per kg body weight. A period of 5 minutes was given to each animal to ensure onset of chemically induced irritation of acetic acid [20], following which period, the number of abdominal constrictions (writhings) was counted for 10 min. The percent inhibitions of abdominal writhings were calculated according to the formula given below.

$$\mathrm{Percent}\;\mathrm{inhibition}=\left(1–W_{e}/W_{c}\right)\;\times \;100$$

where W_e_ and W_c_ represents the number of writhings in aspirin or MEAAS administered mice (Groups 2–7), and control mice (Group 1), respectively.

### Acute toxicity test

Acute toxicity test was conducted as previously described [21]. Mice were divided into nine groups, each group consisting of six animals. Group 1 was given 1% Tween 80 in normal saline (2 ml per kg body weight). The other eight groups (Groups 2–9) were administered, respectively, 100, 200, 300, 600, 800, 1000, 2000 and 3000 mg of MEAAS per kg body weight. All animals were closely observed for the next 8 hours to notice any behavioral changes or mortality and were kept under close observation for the next two weeks.

### Statistical analysis

Experimental values are expressed as mean ± SEM. Independent Sample t-test was carried out for statistical comparison. Statistical significance was considered to be indicated by a p value < 0.05 in all cases [20].

### Preliminary phytochemical screening

Preliminary phytochemical analysis of MEAAS for presence of saponins, tannins, alkaloids, and flavonoids were conducted as described before [22].

## Results

### Preliminary screening of phytochemicals

Various tests conducted for presence of phytochemicals in MEAAS indicated the presence of tannins, alkaloids, and flavonoids.

### Toxicity evaluation

The crude extract did not show any toxicity in mice even at the highest dose tested.

### Antihyperglycemic activity evaluation results

MEAAS, when administered at doses of 50, 100, 200 and 400 mg per kg body weight, dose-dependently and significantly reduced the concentration of blood glucose in glucose-loaded mice by 22.9, 30.7, 45.4, and 46.1% respectively. By comparison, a standard antihyperglycemic drug, glibenclamide, when administered to mice at a dose of 10 mg per kg body weight, reduced blood glucose level by 48.9%. The results are shown in Table 1 and indicate that at the highest dose of 400 mg, the antihyperglycemic activity of MEAAS was comparable to that of glibenclamide.

**Table 1:** **Effect of crude methanol extract of *A. sessilis *** **aerial parts (MEAAS) on blood glucose level in hyperglycemic mice following 120 minutes of glucose loading**

**Treatment**	**Dose (mg/kg body weight)**	**Blood glucose level (mmol/l)**	**% lowering of blood glucose level**
Control	10 ml	5.60 ± 0.27	**-**
Glibenclamide	10 mg	2.86 ± 0.26	48.9*
(MEAAS)	50 mg	4.32 ± 0.16	22.9*
(MEAAS)	100 mg	3.88 ± 0.24	30.7*
(MEAAS)	200 mg	3.06 ± 0.11	45.4*
(MEAAS)	400 mg	3.02 ± 0.17	46.1*

All administrations were made orally. Values represented as mean ± SEM, (n = 5); ^*^P < 0.05; significant compared to hyperglycemic control animals.

### Analgesic activity evaluation results

Dose-dependent and significant reductions in the number of abdominal writhings induced by intraperitoneal administration of acetic acid were observed with MEAAS. At doses of 50, 100, 200 and 400 mg per kg body weight, MEAAS reduced the number of writhings, respectively, by 27.6, 37.9, 41.4, and 44.8%. A standard non-narcotic analgesic drug, aspirin, when administered to experimental animals at doses of 200 and 400 mg per kg body weight, reduced the number of writhings by 31.0 and 51.7%, respectively. Thus the three higher doses of MEAAS exhibited greater analgesic activity than aspirin when administered at a dose of 200 mg per kg body weight. The results are shown in Table 2.

**Table 2:** **Analgesic effect of crude methanol extract of *A. sessilis *** **aerial parts (MEAAS) in acetic acid-induced pain model mice**

**Treatment**	**Dose (mg/kg body weight)**	**Mean number of abdominal constrictions**	**% inhibition**
Control	10 ml	5.8 ± 0.37	-
Aspirin	200 mg	4.0 ± 0.84	31.0*
Aspirin	400 mg	2.8 ± 0.37	51.7*
(MEAAS)	50 mg	4.2 ± 0.49	27.6*
(MEAAS)	100 mg	3.6 ± 0.51	37.9*
(MEAAS)	200 mg	3.4 ± 0.40	41.4*
(MEAAS)	400 mg	3.2 ± 0.37	44.8*

All administrations (aspirin and extract) were made orally. Values represented as mean ± SEM, (n = 5); *P < 0.05; significant compared to control.

## Discussion

The observed decrease in blood glucose levels by MEAAS can be possibly through potentiating pancreatic insulin secretion or by increasing glucose uptake. Such mechanisms have been proposed before for extracts of *Picrorrhiza kurroa*[17] and *Helicteres isora*[23]. Although identification of phytochemicals was not conducted in this preliminary study, flavonoids or alkaloids present in MEAAS can account for the observed antihyperglycemic effect. Free radical scavenging activity as well as antihyperglycemic and antihypertensive effects has been reported for flavonoid-rich fractions from *Trichilia emetica* and *Opilia amentacea* in animal model of type II diabetes mellitus [24]. Hypoglycaemic and tissue-protective effects of the aqueous extract of *Persea americana* seeds on alloxan-induced albino rats has been reported; alkaloids and flavonoids were present among the extract [25].

Intraperitoneal administration of acetic acid can lead to pain (with consequent abdominal writhings) by inducing the release of mediators like prostaglandin E2, as well as lipooxygenase products [26]. Prostaglandins [mainly prostacyclines (PGI_2_) and prostaglandin- (PG-E)], in turn, has been shown to be responsible for excitation of Aδ-nerve fibers, leading to the sensation of pain [27,28]. Thus the observed non-narcotic analgesic activity of MEAAS can be due to its ability to block prostaglandin synthesis through inhibition of lipooxygenase and/or cyclooxygenase activities. A similar mechanism has been proposed before for analgesic activity of *Ficus deltoidea* aqueous extract in acetic acid-induced pain model [26]. Flavonoids, alkaloids and tannins present in MEAAS can be responsible for the analgesic effect. Flavonoids, alkaloids and tannins have been shown to be present in methanol extract of *Muntingia calabura* leaves demonstrating analgesic activity [29,30].

The present study validates the folk medicinal use of *A. sessilis* in Bangladesh for treatment of severe pain, and further suggests that the aerial parts of the plant can be a potential mean for lowering blood glucose levels. Since the plant is widely available in Bangladesh, it can prove beneficial in being a source of a cheap and effective medication for persons with high blood glucose levels and persons suffering from chronic pain in cases like rheumatoid arthritis. Notably, the plant is consumed by the people of Bangladesh as a vegetable. Further studies are underway in our laboratory as to whether cooking destroys the antihyperglycemic and analgesic principles or the principles still remain active.

## Conclusion

The results validate the folk medicinal use of stems of *A. sessilis* to reduce high blood glucose levels in diabetic patients and to alleviate pain. From that view point, the extract merits further scientific attention for further isolation and identification of the responsible bioactive component(s).

## Competing interests

The author(s) declare that they have no competing interests.

## Authors’ contributions

AIH, MF and SR collected the plant, did the extraction, and performed the experiments under the supervision of RJ and MR. MR wrote the manuscript draft, which was read and edited by all authors. All authors read and approved the final version of the manuscript.

## Pre-publication history

The pre-publication history for this paper can be accessed here:

http://www.biomedcentral.com/1472-6882/14/169/prepub
